# Detour canal, a civil engineering heritage created through historical struggle for water resources, now provides the habitats for a rare freshwater fish

**DOI:** 10.3897/BDJ.12.e119517

**Published:** 2024-06-27

**Authors:** Yohei Yamasaki, Hironori Hayashi, Suguru Kubo, Takashi Namiki, Yuichi Kano

**Affiliations:** 1 Kyushu University, Fukuoka, Japan Kyushu University Fukuoka Japan; 2 WWF Japan, Tokyo, Japan WWF Japan Tokyo Japan; 3 Kyushu Open University, Fukuoka, Japan Kyushu Open University Fukuoka Japan

**Keywords:** civil engineering heritage, irrigation ditch, rare species, fish biodiversity, conservation ecology, *Tachysurusaurantiacus*, *Pseudobagrusaurantiacus*

## Abstract

The Ariake catfish, *Tachysurusaurantiacus*, is a freshwater fish endemic to Kyushu Island, Japan. However, these catfish are now endangered owing to environmental changes. Despite their status, there is scant quantitative research on the Ariake catfish regarding their potential conservation. The Yabe River is a typical catfish habitat situated in the northern part of Kyushu Island (Ariake Area) and has a unique civil engineering heritage, as represented by the so-called ‘detour canal’. The canals were created owing to competition by two Domains to divert additional water resources into their own territory for rice cultivation during the Edo Period (1603–1867). To fill the research gap on the Ariake catfish and assess the ecological value of detour canals, in this study, we conducted a survey of local catfish populations and nine environmental parameters that can affect them. We found that the population volume of the Ariake catfish was significantly higher in canals than in ordinary branch rivers. Although the detour canals were not originally constructed for biodiversity conservation, they nonetheless unintentionally provide catfish habitat at present. As these canals represent a remarkable example of a contribution by a civil engineering heritage structure to biodiversity conservation, our study should be used as a potential justification for preserving the canals, as well as conserving the aquatic species that utilise them as vital habitat.

## Introduction

Japan is a biodiversity hotspot (Mittermeier et al. 2004) and is home to numerous freshwater fish, including endemic species. However, many species are facing extinction crises due to a variety of factors, such as habitat destruction and invasive alien species (The Ichthyological Society of Japan 2016).

The Ariake catfish (or Ariake cuttailed bullhead), *Tachysurusaurantiacus*, is a freshwater fish endemic to Kyushu Island, Japan (Hosoya 2016). These catfish are generally found in slow-flowing areas in the middle reaches of rivers in alluvial fans and are nocturnal; moreover, they hide in vacant spaces, such as submerged vegetation and stream bed rocks/stones (Hosoya 2016). The catfish is endangered and listed as ‘Near Threatened (NT)’ (Miyazaki et al. 2019) or ‘Vulnerable (VU)’ (Ministry of the Environment of Japan 2020). There are several possible reasons for this decline: habitat destruction by river modification and fragmentation by weirs are potential negative factors (Hosoya 2016, Miyazaki et al. 2019), but no objective data are available on this subject. Moreover, no quantitative studies have been conducted on catfish from the perspective of ecological conservation.

The Yabe River, located in the northern part of Kyushu Island (Ariake area), has a basin of 618 km^3^, a total length of 61 km and an irrigated field of 14,100 ha. The Yabe River is one of the most biodiverse rivers in Japan, with more than 80 freshwater fish species, of which 30 are important/rare species (Ministry of Land, Infrastructure, Transport and Tourism of Japan 2023). The Ariake catfish also inhabits the river and is a symbolic fish of the area as signified by its moniker.

**Figure 1.**
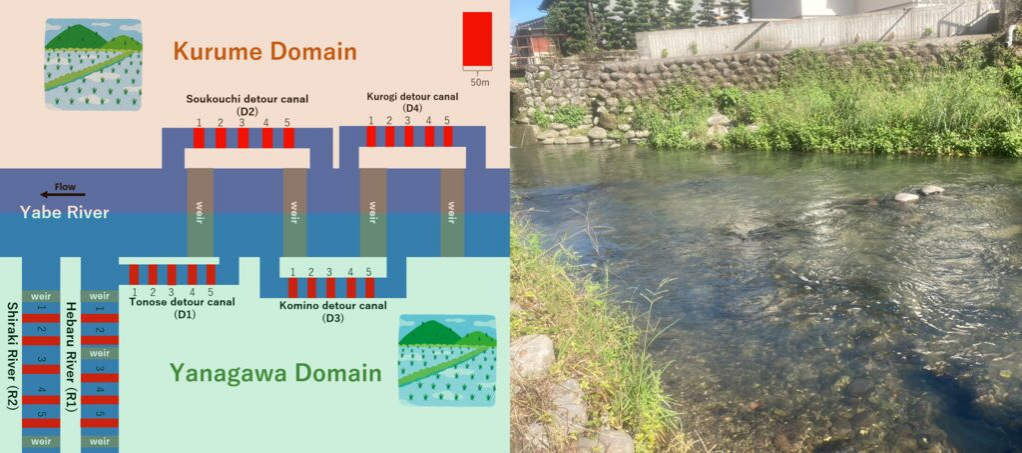
Left: Diagram of the detour canals constructed as the result of a historical water struggle between the Kurume and Yanagawa Domains. Red parts show the study sections. Right: A photograph of a detour canal.

Historically, the irrigation system of the Yabe River has been unique, as it contains ‘detour canals’ that constitute civil engineering heritage structures. These canals were constructed during the Edo period (1603–1867) as water-management facilities for irrigation (Mashino 1997, Baba 2007). In detail, during the Edo period, the Yabe River was at the border between the Kurume and Yanagawa Domains. Both domains required water for rice production and, therefore, competed for additional water resources. Thus, each domain constructed ‘detour canals’ to bypass the water into its own territory (Fig. 1). Detour canals are still in use today approximately 300 years after their construction and they exhibit physical characteristics that distinguish them from ordinary river canals. For example, detour canals are less susceptible to destruction by floods and the impact of artificial modifications is smaller. Consequently, a stable aquatic environment has been maintained within these canals over time.

Many civil engineering heritage sites provide flood control and water utilisation capabilities and are highly compatible with the local landscape and culture. Furthermore, previous studies have shown that they have a positive impact on ecosystems (Nagaya et al. 2017, Tazumi et al. 2019). To highlight this impact, the present study focuses on how historical civil engineering heritage contributes to biodiversity conservation. Moreover, we attempt to fill the existing quantitative research gap regarding potential Ariake catfish conservation.

## Materials and Methods

### Study site

Of the seven existing detour canals in the Yabe River, four canals belonging to the alluvial fan ecoregion (Itsukushima and Shimatani 2016) were the focus of this study. The detour canals were as follows: Tonose (D1), Sokouchi (D2), Komino (D3) and Kurogi (D4). In addition, two branches of the Yabe River that also reside within the alluvial fan eco-region and have similar flow conditions and scales to the canals, namely the Hebaru River (R1) and Shiraki River (R2), were included as controls in this study. Five study sections of 50 m in length were selected at approximately equal intervals in each canal/river (Fig. 1). A field survey was conducted between October and December 2022.

### Fish sampling

The number of Ariake catfish in each section was recorded by three individuals using an electric shocker (LR-24, Smith-Root Inc., Vancouver, WA, USA) and a hand net. One person carried the electric shocker, one person collected the catfish with a hand net and another person counted the catfish and promptly released them after recording. The sampling effort was 30 min per section along a 50 m stretch.

### Environmental parameters

Nine environmental parameters were selected for this study that potentially affected the population volume of catfish: 1) The most important parameter in this study was to discriminate between detour and non-detour canals. Dummy variables were used to perform the discrimination; 2) The coverage rate for bankside vegetation (littoral submerged macrophyte cover), which may function as a fish refuge, was estimated by manually sketching the sectional environment; 3) We also measured the coverage rate of water-based vegetation (not on the bank, but inside the water flow) using sketches, as aquatic plants such as *Hydrillaverticillata* and *Potamogetoncrispus* sometimes covered a part of the water bottom; 4) The concrete revetment rate was measured using a sketch; parts of the canals and rivers were artificially riveted by concrete, which could potentially ecologically affect the catfish; 5) Substrate (rock/stone/pebble) sizes were measured at 50 equally spaced locations in the section and averaged; a larger substrate can potentially provide habitat for Ariake catfish; 6) The water velocity at 60% of the water depth was measured at 50 equally spaced locations in the section and averaged; 7) The water depth was measured at 50 equally spaced locations in the section and averaged; 8) The elevation at the water surface level was measured using RTK-GNNS (R4 GPS VRS Bundle, Trimble Inc., Colorado, USA) at the centre of the section; 9) The gradient of each section was calculated as the difference between the water-surface elevation at the uppermost upstream point and that at the lowermost downstream point.

### Statistical analysis

A generalised linear mixed model (GLMM) analysis was conducted to clarify the relationship between the nine environmental parameters and the Ariake catfish population volume. The number of catfish collected from each study section was used as the response variable. In contrast, the detour canal, bank-side vegetation rate, water vegetation rate, concrete revetment rate, substrate size, water velocity, water depth, elevation and gradient were used as explanatory variables.

Statistical software R (ver. 4.2.3) with the ‘glmer’ function of the ‘lme4’ package was used to perform the analysis. Data were clustered into the respective canals/rivers as a random effect of the GLMM. A Poisson distribution was used as a log-link function. The Akaike Information Criterion (AIC) was used to select significant variables and obtain the best 10 models.

## Results

### Fish sampling and environmental parameters

**Figure 2.**
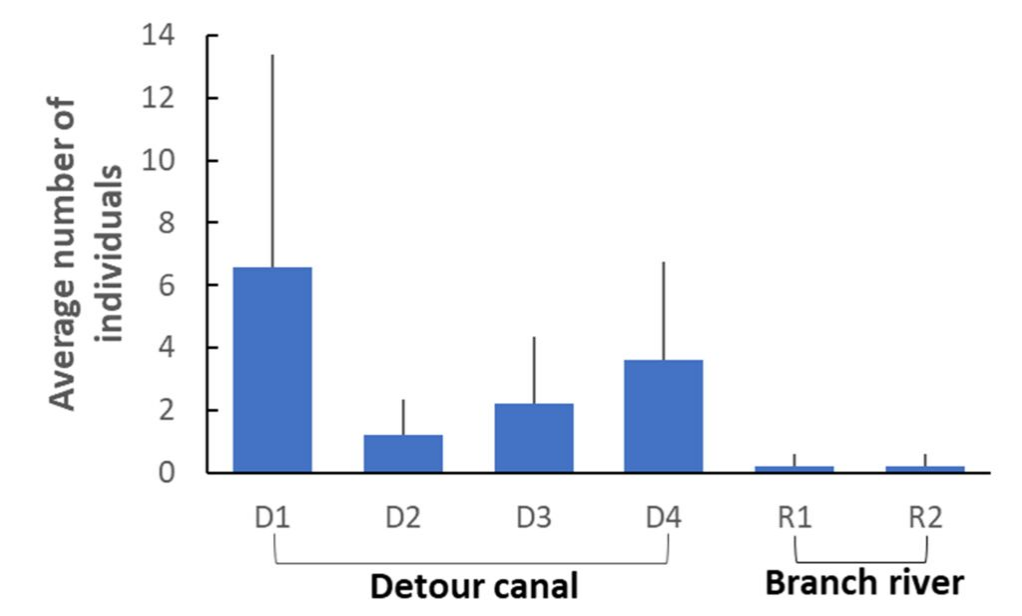
Average individual number of Ariake catfish per 50 m section in each canal/river with an image of the species. The error bar shows the standard deviation.

As a result of the fish sampling survey, 70 individuals (approximately 20–200 mm in total length) were collected from detour canals and branch rivers: 33 in D1, 6 in D2, 11 in D3, 18 in D4, one in R1 and one in R2 (Fig. 2).

The environmental parameters of the canals and rivers are shown in Suppl. material 1.

### Statistical analysis

The results of the GLMM analysis are presented in Table 1. The detour canal had a significant positive effect on the volume of the catfish; the variable was selected in all the best 10 models and the P-values were all < 0.001. Bankside vegetation also showed a significant positive effect on catfish, as selected in all best 10 models with highly significant P-values. However, the water vegetation rate was not selected for any of the models. The concrete revetment rate was selected as a positive factor in the low-ranked models, although none had significant P-values. The substrate size was selected in the middle-ranked models, with no significant P-values. Water velocity was selected as a positive factor in three models and the lowest model had a significant P-value. The water depth was not selected for any of the models. Elevation was selected as a positive factor in the four models without significant P-values. This gradient was selected in five models, although there were no significant P-values.

## Discussion

The detour canals provided an essential habitat for the Ariake catfish (Fig. 2); 68 of the 70 individuals were caught in the detour canals. The GLMM statistical analysis showed a highly positive effect of the detour canal on catfish populations (Table 1). The detour canals had conditions suitable for catfish inhabitation. Detour canals are not rivers, but agricultural canals are artificial infrastructure. Thus, the sluice gates were closed during main river floods. The intensity of the flood disturbance of the detour canals was much lower than that of the main and branch rivers and the aquatic environment has been maintained stably since its establishment to the present day. Additionally, compared to the main river and branch rivers, which were repaired every time they were damaged by floods, detour canals have have been subjected only to a few human modifications until recently. The lower impact of anthropogenic change is likely advantageous for catfish and other species and ecosystems.

Furthermore, although the detour canals were constructed for drawing water into an historical domain, they now function as a continuous connection between the upper and lower reaches of the Yabe River beyond the weirs (Fig. 1). This implies that detour canals act as fish passageways, contributing to the ecological continuity and networks of rivers. However, by contrast, the branch rivers have weirs, but no detour canals (Fig. 1), which block fish migration. Hosoya (2016) and Miyazaki et al. (2019) suggested that network fragmentation is a negative driver of catfish decline and our results support this indication.

Bankside vegetation was another significant positive factor affecting catfish populations (Table 1). The complex structures and spaces provided by submerged macrophytes likely function as habitats for catfish (Hosoya 2016). Many studies have indicated the importance of riverbank vegetation for fish biomass and assemblages (e.g. Collares-Pereira et al. (1995), Randall et al. (1996), Smokorowski and Pratt (2007)). However, aquatic vegetation (inside the river) was not an important factor (Table 1). We suspect that pure aquatic plants, such as *H.verticillata* and *P.crispus*, are excessively dense and tender and, thus, cannot provide space for catfish.

In conclusion, the biodiversity conservation value of detour canals has been clarified through our findings. The detour canals are valuable in their own right as civil engineering heritage sites, but their ecological function of providing habitat for the rare Ariake catfish species can be clearly stated. In this study, we focused only on Ariake catfish and we suspect that many other aquatic species may benefit from detour canals.

Unfortunately, the social outlook for detour canals is not optimistic. Owing to the aging of canals and requests from local populations, renovating canals with hard concrete is spreading in some areas. These modifications would degrade the ecological function of the canals and cause the loss of the local cultural landscape (Fig. 1). We hope that the findings of this study will lead to a review of the value of detour canals and contribute to the maintenance of catfish habitats and, eventually, the conservation of biodiversity (WWF International 2022) and culturally important sites.

## Supplementary Material

EA5D537D-4ADD-5EA7-B7DC-C77BCC65EEFE 10.3897/BDJ.12.e119517.suppl1 Supplementary material 1 Environmental parameters of each section Data type csv Brief description The nine environmental parameters of each section. Standard deviation is shown in parentheses. File: oo_962899.csv https://binary.pensoft.net/file/962899 Yohei Yamasaki

## Figures and Tables

**Table 1.** Coefficients of the best 10 GLMM models that explain the individual number of Ariake catfish according to AIC.

Model	AIC	Detour canal (1/0)	Bankside vegetation rate	Water vegetaion rate	Concrete revetment rate	Substrate size (m)	Water velocity (m/s)	Water depth (m)	Elevation (km)	Gradient (‰)
1	131.6	3.24***	2.24***							
2	131.6	2.96***	2.56***				2.10		14.4	-0.079
3	131.9	3.27***	1.89***			0.49				-0.071
4	132.0	3.17***	2.56***						9.85	
5	132.1	3.82***	3.17***		1.09	0.52				
6	132.4	3.62***	2.71**		0.88	0.66				-0.061
7	132.5	3.43***	2.22***			0.29				
8	132.5	3.06***	2.07***							-0.043
9	132.6	3.52***	3.70***		0.99		2.15		11.8	
10	132.6	3.19***	3.24***		0.69		2.58*		13.8	-0.068

*P < 0.05, **P < 0.01, ***P < 0.001
